# A Qualitative Study of Patients' Attitudes toward HIV Testing in the Dental Setting

**DOI:** 10.1155/2012/803169

**Published:** 2012-02-16

**Authors:** Nancy VanDevanter, Joan Combellick, M. Katherine Hutchinson, Joan Phelan, Daniel Malamud, Donna Shelley

**Affiliations:** ^1^NYU College of Nursing, 726 Broadway, New York, NY 10003, USA; ^2^NYU College of Dentistry, 345 E. 24th Street, New York, NY 10010, USA

## Abstract

An estimated 1.1 million people in the USA are living with HIV/AIDS. Nearly 200,000 of these individuals do not know that they are infected. In 2006, the CDC recommended that all healthcare providers routinely offer HIV screening to adolescent and adult patients. Nurse-dentist collaborations present unique opportunities to provide rapid oral HIV screening to patients in dental clinic settings and reach the many adults who lack primary medical providers. However, little is known about the feasibility and acceptability of this type of innovative practice. Thus, elicitation research was undertaken with dental providers, students, and patients. This paper reports the results of qualitative interviews with 19 adults attending a university-based dental clinic in New York City. Overall, patients held very positive attitudes and beliefs toward HIV screening in dental sites and identified important factors that should be incorporated into the design of nurse-dentist collaborative HIV screening programs.

## 1. Introduction

An estimated 1.1 million people are currently living with HIV in the USA, yet one in five are unaware of their status [1]. Early diagnosis and treatment have dramatically increased lifespan and reduced HIV transmission; yet 55% of adults, ages 18–64, report they have never been tested for HIV. Among those who have been tested and were found to be infected, at least one-third discovered their status late in the course of their illness, thus missing the opportunity to receive the maximum benefits that early treatment provides [1].

To facilitate early diagnosis and treatment, the Centers for Disease Control and Prevention (CDC) issued revised recommendations in 2006 for widespread HIV testing [2]. The new recommendations proposed that HIV testing be offered to all individuals, ages 13 to 64, in all healthcare settings on an “opt-out” basis, rather than waiting for patients to request testing. These revisions brought HIV testing in line with other STI protocols and helped reduce what had been referred to as “HIV exceptionalism” [3]. Requirements that were specific to HIV testing that were often viewed as barriers to expanded screening, for example, written informed consent, were eliminated [2]. In addition, the CDC recommended that prevention counseling no longer be required. These revised standards, along with the availability of rapid testing technology, have helped bring HIV screening to new venues [4–6].

Dental practice sites represent new venues with great promise [6–8] and the potential to reach millions of Americans who see a dentist but not a physician annually [4, 5, 9, 10]. Although HIV testing may not appear, on the surface, to be within the scope of dental practice, the development of rapid oral fluid diagnostic test kits for HIV provides the opportunity for oral screening to become a logical extension of routine dental care [4, 5]. However, to date, few dentists and few academic dental centers have incorporated HIV screening into practice [5].

The American Academy of Nursing Expert Panel on Emerging and Infectious Diseases (AAN Expert Panel) posited that providers' perceived barriers to HIV testing might be inhibiting uptake into practice in a variety of healthcare settings [11]. However, there is a dearth of research that has examined the factors that promote or inhibit HIV screening in dental practice sites. One study conducted with dental educators found that, although the majority support offering HIV screening in the dental setting, only a small percentage actually do so [5]. A recent study with dental faculty and students [7] found that, consistent with the 2010 AAN Expert Panel report [11], there were a number of negative provider perceptions acting as barriers to HIV testing [7]. Consistent with the findings from an earlier national study [5], dental providers' reported barriers to HIV testing included perceived lack of skills in HIV testing and counseling, lack of appropriate referral sources and logistical issues such as time, cost, and patient privacy [7].

Many of these potential barriers to HIV testing could be addressed through collaborations between nurses and dental providers [7, 8]. Since early in the AIDS epidemic, nurses have played an important role in the provision of HIV testing and counseling [12]. The 2010 American Academy of Nursing Expert Panel endorsed widespread HIV testing and advocated for interdisciplinary partnerships between nurses and other healthcare providers in order to facilitate the implementation of the CDC recommendations for widespread HIV testing in all healthcare settings [11]. Although there is little precedent for dental-nursing collaboration in general, in the field of HIV/AIDS care, nurses have become knowledgeable about oral manifestation of HIV despite the lack of much formal training in oral health assessments [12]. Formal training by dentists to conduct comprehensive oral examinations could enhance early detection of oral health-related problems by nurses. Similarly, dentists can benefit from nurses' knowledge about the management of potential patients concerns about HIV testing and counseling and referral of those who screen positive.

In addition to concerns about personal skills and logistical issues, some dentists and dental students have voiced concerns that patients would react negatively to offers of HIV testing [7]. However, these findings were limited to the perspectives of dentists and dental students. Few studies have examined patients' actual beliefs and perceptions regarding HIV testing in the dental setting. Following implementation of the revised CDC HIV testing recommendations in 2006, several studies found high levels of patient acceptability of HIV testing in nondental healthcare settings, including emergency departments [13], and outpatient clinics [14, 15]. Two studies found similarly high levels of acceptability of HIV testing among dental patients [6, 16]. In 2007, a patient survey conducted at an urban, free dental clinic in Kansas City Missouri found that 73% of 150 participants stated they would accept free HIV screening at their dental visit [6]. Most of the participants were African American females who resided in neighborhoods with high HIV prevalence; 61% had previously been tested for HIV. Among those who had never been tested, 74% stated that they would agree to be tested if offered in the dental setting. Most participants had no specific preference as to who should provide HIV testing results and only 11% suggested that it should be a counselor trained in HIV counseling. Reasons for refusal included pain due to dental procedures, concerns about HIV stigma from providers and others, accuracy of the rapid HIV test, and time constraints [6]. In 2008, a counselor-based rapid HIV (blood) testing program was implemented in the Harlem Hospital dental clinic. More than 97% of the 3,565 individuals who were offered free testing accepted [16].

While encouraging, these results reflected experiences in single dental practice sites. They also did not provide the depth of understand of patients' beliefs, perceptions, and site-specific barriers necessary to guide the development of a collaborative nurse-dentist rapid oral fluid HIV screening program at a large, urban, university dental center. Thus the purpose of this study was to examine dental patients' attitudes, beliefs, and perceived barriers to HIV screening in order to address these factors in an implementation plan.

## 2. Materials and Methods

Data from patients were collected as part of a larger pilot study assessing the feasibility of implementing routine HIV screening and counseling in a large university-based dental clinic. In-depth interviews were conducted with 19 new patients between April and May of 2011 to assess their attitudes, beliefs, and perceived acceptability of rapid oral HIV testing in the dental clinic setting.

### 2.1. Setting and Participants

 The study took place at the NYU College of Dentistry (NYUCD), the single largest provider of low-cost dental care in New York state. NYUCD serves as an essential safety net for the underserved, uninsured, underinsured and other marginalized and at-risk residents of New York City and its surrounding areas. In 2010, the clinic served more than 82,000 new patients and provided over 383,000 clinic visits, 41% came from the 30 New York City area Zip Codes with the highest HIV seroprevalence rates. Among NYUDC patients 74% are between the ages of 30 and 49, slightly more than half (54%) are female, about 60% are Black or Latino, 40% are uninsured, 55% Medicaid insured, and 5% have private insurance. Prior to initiating the study, all of the human subjects-related documents and procedures were approved by the appropriate IRB at New York University. Although all potential participants were given a copy of the written informed consent along with contact information of the principal investigator, signed informed consent forms were not collected as these would have been the only participant identifiers.

### 2.2. Procedures

A convenience sample of study participants were recruited from patients who were registering for care at the NYUCD Admissions Clinic during afternoon sessions on variable week days. Patients were approached in the waiting room by a trained and experienced research assistant and invited to participate in the study (complete an interview). Potential participants were given an information sheet/invitation to participate that explained the purpose of the study as well as the incentive offered (a $20 New York City transit card). Those who agreed to participate were taken to a quiet private area for interviews.

### 2.3. Data Collection and Analysis

A patient interview guide was used that focused on eliciting patients' beliefs and attitudes and intentions regarding HIV screening in the dental clinic. Participants were also asked for their perceptions of factors that would make it easier (facilitators) and those that would make it more difficult (barriers) to be screened for HIV in the dental setting. Interviews lasted between 15 and 25 minutes and were audiotaped. Audiotapes were subsequently transcribed verbatim and qualitative thematic content analysis [17, 18] was conducted by two investigators and a graduate public health student to develop a preliminary coding scheme. The coding scheme facilitated the systematic identification of analytic patterns that became apparent from the data, as well as theoretically important concepts. Limited demographic information was obtained from participants.

Eighteen of the nineteen interviews were conducted in English. One interview was conducted in Spanish because the bilingual participant requested Spanish. All participants were cognitively functional and medically stable and were over the age of 18.

## 3. Results

About three quarters of participants were between the ages of 30 and 49; 58% were female. More than 50% were Caucasian, 22% Latino, and 11% African American. Almost half were unemployed, 53% were Medicaid insured, 22% had no insurance, and 22% had private insurance. Almost one-third stated they had no primary care provider; however about 90% reported seeing a healthcare provider in the last year. Only 42% reported having been previously offered oral HIV testing by a medical provider.

Analysis of the interview transcripts revealed three main themes related to patients' views on rapid oral HIV testing in the dental setting: (1) acceptability and perceived advantages to rapid oral HIV testing in the dental setting, (2) congruence between HIV screening and patients' views of dental settings and the role of dentists, and (3) logistical issues related to implementation of rapid oral HIV testing in the dental setting.

### 3.1. Theme 1: Acceptability and Perceived Advantages to HIV Testing in Dental Settings

All study participants expressed very positive attitudes toward rapid oral HIV testing in the dental setting for themselves and for other patients. Almost three-quarters (74%) said they would accept screening if it were offered as part of the dental visit. A variety of reasons were offered. Some thought that it was important for people to *know* their HIV status as the following statements illustrate: “I think that is terrific…because people might have it—might be a carrier and not know it” (R1); “It's a good thing…there's no risk involved, there's a lot of people out there that would want to be checked” (R8).

Other participants stressed the *convenience* of getting HIV testing in the dental environment: “It would be a great idea because…there is never enough time to check my HIV status” (R17); “I'm gonna be laid back getting my teeth done, I might as well—two birds with one stone” (R14); and “It might be a perfect time, because it's through the mouth” (R19). One participant responded enthusiastically, “Wow, this is a very good idea. I came to the dentist and they did the test. Better than when you have to go to a particular place and wait a long time” (R6).

Two of the advantages identified by participants were that the test would be offered *free of charge *and *universally. *One participant explained, “for people like myself who can't afford insurance, aren't in a stable position, [they can't] go somewhere else and get it done” (R4). Another said an incentive for him was “it's cheap, it's free” (R13). Most participants said that everyone should be offered testing and most said that it should be voluntary. One participant noted the disadvantage of specific patients being offered testing saying, “It should be offered to everyone who comes…if you pigeon hole it, then people are going to say, well you're acting in a racist manner, social Darwinistic manner” (R19).

When participants were asked why some patients might not accept rapid oral HIV testing, responses included: *fear* of getting a positive HIV test result, for example, “Some people are afraid of the results” (R1);* ignorance*, for example, “People aren't really educated about HIV or know what it is about…they do not want to be anywhere near it, around it, talk about it. It's not going to happen to me”; *prior knowledge*, for example, “They might know they have it.” Several stated that older patients might not recognize the importance of testing because of low self-perceived risk for HIV infection.

When asked whether other patients might be offended at being offered rapid oral HIV testing in the dental setting, one responded, “I think a lot of Caucasian folks who are middle class they would be adverse to it” (R9). However, others noted, “[HIV] has been [around] quite a few years…things have changed” (R1), and “we live in a huge metropolitan city…most people are at least aware” (R4).

### 3.2. Theme 2: Congruence of HIV Screening and Patients' Views of Dental Settings

Participants were very positive about being offered rapid oral HIV testing in the dental clinic setting and thought it consistent with their view of dental practice. One participant stated, “I strongly believe that, if a place like this is offering testing, it's only for the good of everybody, for patients as well as doctors. It totally shows me that this place cares about peoples' health” (R18). Another noted that patients might initially be surprised about being offered rapid oral HIV testing but thought it was a good idea: “It's a good thing…I'd probably be surprised just because it wouldn't occur to me that a dentist would offer that but I think as long as there was some sort of counseling available…It would be a surprise factor, not really expecting this…you expect to be asked about your oral history, not necessarily your sexual history” (R4). Others expressed no surprise at the idea because the testing was by oral swab: “It kind of goes together, the teeth, the mouth, the dentist” (R19); “But with the swab, I think because it's an oral thing, I can see why that makes sense” (R4). Other participants saw dental care as an integral part of medical care as the following statements reflect: “I believe dental care is medical care” (R18); “I feel they are so closely related [medicine and dentistry] that it's not like we're going to the grocery store to get tested” (R18); “I don't see any difference between a dentist or a doctor” (R7). In addition, one participant suggested that in some circumstances a patient may go to a dentist but not necessarily a doctor, “Sometimes people don't go to doctors but if they have a toothache, they will go to a dentist” (R5).

### 3.3. Theme 3: Logistical Issues Related to Implementation

Participants identified a number of logistical issues related to implementation of rapid oral HIV testing in the dental practice setting, including getting positive test results; need for professional counseling and linkage to care for HIV-positive patients, providing HIV prevention educational materials and the need for privacy.

Most participants raised concerns about how they or another patient might feel about learning they have a positive test result and their ability to deal with those emotions. As one participant explained, “I would be stressed out” (R3). About one-third stated they believed there was a need for professional counseling for HIV-positive patients in dental settings: “you need someone to deal with the psychosocial effects” (R2). Another said, “I think how you receive the news is very important…I wouldn't want to hear it from a dental student” (R4). Another pointed out the stress this would create for the dental provider as well as the patient: “I'd be concerned that there wouldn't be anyone there to really work with the person if they did get a positive or even if it was a false positive. I don't want to speak for dentists but I'm sure it's stressful but nowhere near as stressful as delivering some pretty devastating news to people” (R17). One participant thought the patient should not be given the results until the confirmatory test was done. Several participants stressed the importance of privacy for getting test results especially in the dental clinic setting where many patients, dental students, and dental faculty are in close proximity.

 When asked about HIV prevention education in the dental setting, participants were unanimous in their support for providing educational materials in the dental clinic but much more cautious about offering free condoms. One said simply, “I would be offended [if someone offered me condoms]” (R1). Another participant suggested that condoms could be offered discretely: “in a bag” (R2); “it's tricky…they should be free and available but if a mother came in with a 16-year-old…” (R4); “I don't think there should be someone at the door handing them out. There should be something on the side of the office with a sign “Practice safe sex, there's condoms. Take one if you want” (R7); “It's a little strange to see them in a dentist's office. You might scare people a little” (R11). Another participant pointed out that some patients might have religious objections to being offered condoms: “handing them out is over the top… you know, maybe they're conservative Catholic, Hasidim, conservative Muslim…that might piss them off” (R9).

## 4. Discussion

Participants reported universally positive attitudes about being offered rapid oral HIV testing in dental settings and expressed beliefs that that the provision of such testing was consistent with their view of the dentist's role. These findings are consistent with dentists' and dental students' perceptions in a recent study [7], as well as two studies of patient acceptability for HIV testing in the dental practice setting [6, 16]. The main benefits patients identified for such testing were increasing individuals' knowledge of their HIV status and the convenience of being tested while they were receiving dental care. While the study did not directly explore the issue of cost, several participants voluntarily mentioned free testing as an important incentive. The two previously published studies of patient perspective on HIV screening in dental setting provided free testing and demonstrated high acceptability [6, 16]. Even though only 22% of participants were uninsured, both insured and uninsured patients voluntarily mentioned cost as an issue, thus, it is likely that reducing financial barriers to HIV testing will increase acceptability. The vast majority of participants felt HIV testing should be offered to everyone on a voluntary basis. The identified barriers to HIV testing for dental patients included fear of receiving a positive result, lack of awareness of HIV, and knowing their HIV status already.

Several challenges to HIV testing in the dental setting were identified by patient participants. Most were concerned about the emotional impact of learning about an HIV-positive test. This concern was not specific to the dental environment but a general concern about the emotional impact of such information. Many participants suggested that, although dental providers could convey an HIV-positive test result to a patient, they should provide timely access to a professional with HIV psychological counseling skills to provide further information and support to patients. Several participants stressed the importance of privacy for receiving test results in this setting. Though they were unanimous in their support for dental professionals providing education to their patients about HIV prevention, many felt that offering condoms to dental patients might offend some patients and recommended a discrete, passive availability of condoms.

Patient concerns about HIV testing in the dental setting in this study differ from those reported by Deitz and colleagues [6] just after the 2006 CDC HIV testing policy changes were released. In that study, patients identified concerns about HIV stigma from dental providers and others, accuracy of the rapid HIV, test and time constraints as important barriers. The absence of patient concerns about stigma and testing accuracy in the current study may reflect changes in public perceptions during the past five years, geographic differences as the current study took place in New York City, and/or the impact of the CDC policy to routinely test for HIV rather than testing on the basis of HIV “risk.”

Further, the data presented here suggest that in order to address patient concerns there is a need to develop detailed protocols for rapid oral HIV testing that address protecting patient privacy, providing professional psychosocial support for patients who receive positive HIV test results, managing referrals and linkages to care for those who test positive, and providing educational materials in the dental practice setting. The present study contributes important information to the small body of literature on the acceptability of HIV testing by dental patients. This is the first qualitative study to examine these issues and provides important contextual information to guide the development of protocols to address patient concerns. The findings support recommendations for the establishment of new interdisciplinary models of care to provide HIV testing in dental settings and to meet the needs of the dental patient with a positive HIV test [7, 8].

## 5. Limitations

These findings should be viewed in light of several study limitations. The study setting is a large urban academic dental clinic in a city with high HIV seroprevalence; adults from suburban and rural areas were not represented among the participants, and their perspectives were not reflected in the data. Although the sample size was small, that was not considered a limitation as data saturation was reached for all themes. Study participants were similar to the general clinic population in terms of age and gender but less likely to be uninsured (22% versus 40%). Since dental services in the USA are largely financed through self-payment rather than through dental insurance, the findings here may not be applicable to the larger uninsured population. Also, fewer than 50% of the participants were from minority racial and ethnic groups, compared to 70% in the larger clinic population. However, in other studies, individuals who were uninsured and members of minority groups at high risk for HIV demonstrated very high acceptability for HIV testing. Thus, the positive attitudes presented here may be similar to those held by those dental patients. Despite this, individuals who held extremely negative attitudes towards HIV testing in the dental clinic may have been less likely to agree to participate. As such, their voices would not have been heard.

## 6. Conclusions

Rapid oral HIV testing in the dental practice setting holds great promise for reaching a proportion of the population not currently accessing primary care in more traditional medical settings. For HIV testing in this setting to be successfully implemented, concerns raised by patients will need to be addressed including testing-related privacy, availability of expert posttest counseling, psychosocial support, follow-up confirmatory testing and linkage to medical care. Collaborations between dentists and other health professionals with specialized training in HIV testing and counseling, such as nurses, provide an innovative approach to address these issues. Such a collaborative approach to HIV testing in a dental clinic setting is currently being developed between the NYU College of Dentistry and the NYU College of Nursing Faculty Practice located at the College of Dentistry. In this model, the dental faculty or dental students will offer testing as part of routine care at the beginning of the oral examination. For patients who accept the offer of testing, dentists/dental students will conduct the oral swab HIV test and the nurse coordinator for the project will report the test results to the patient in a private setting at the end of the visit. For patients whose test is reactive the nurse will provide posttest counseling and linkage to care under the supervision of the NYU NFP. The colocation of these programs offers seamless continuity of care and linkage to specialized care for patients who test positive for HIV [8]. Although most dental practices do not have this kind of co-located access to nurses or other medical providers, developing collaborative relationships with nurse practitioners in the local community for purposes of HIV testing and referral offers a potential solution for dentists in small or solo practices. Although there are no scope of practice issues for dentists or nurses in performing oral diagnostic tests, insurance reimbursement mechanisms for HIV testing and other oral diagnostics in collaborative models such as this will need to be addressed.
